# Supplementary material for: Hippo Signaling Regulates High-NaCl-Induced Increase in RORγt+ Pro-Inflammatory Lymphocytes
Source: Int J Mol Sci. 2025 Feb 27;26(5):2143. doi: 10.3390/ijms26052143 (PMC11900413; doi:10.3390/ijms26052143)

Supplementary Information

Supplementary table S1

Ratio of regularized logarithmic expression of genes between Th17/ Treg and Th17/ naive T cells (7)

gene_symbol	Full name	Th17/naive	Th17/Treg
Abp1	Amiloride-sensitive amine oxidase [copper-containing]	47.80	2.62
Ak1	Adenylate kinase isoenzyme 1	4.87	2.49
Akr1b8	Aldose reductase-related protein 2	7.24	2.61
Akr1c18	Aldo-keto reductase family 1 member C18	5.78	2.86
Ank2	Ankyrin-2	2.74	7.08
Ankk1	Ankyrin repeat and protein kinase domain-containing protein 1	2.40	2.12
Atp1a2	Sodium_potassium-transporting ATPase subunit alpha-2	2.01	2.04
Bdh2	Dehydrogenase_reductase SDR family member 6	8.99	2.83
Ccl20	C-C motif chemokine 20	13.70	3.67
Cd40	Tumor necrosis factor receptor superfamily member 5	3.20	2.03
Cnih3	Protein cornichon homolog 3	3.90	4.26
Ctgf	CCN family member 2	3.77	3.70
Cyp1a1	Cytochrome P450 1A1	3.98	2.73
Cyr61	Dehydrogenase_reductase SDR family member 9	2.49	2.43
Dhrs9	Dystrobrevin alpha	9.81	2.84
Dtna	RNA polymerase II elongation factor ELL3	3.05	3.49
Ell3	Ephrin type-A receptor 5	2.53	7.08

gene_symbol	Full name	Th17/naive	Th17/Treg
Epha5	Fermitin family homolog 1	3.27	3.86
Fermt1	Fibrinogen-like protein 1	5.22	3.88
Fgl1	Frizzled-9	2.68	2.60
Fzd3	Frizzled-3	2.15	2.01
Gatm	Glycine amidinotransferase, mitochondrial	6.32	2.05
Gcm1	Chorion-specific transcription factor GCMa	3.18	5.01
Gdf3	Growth_differentiation factor [illegible]	2.92	2.68
Gng11	Guanine nucleotide-binding protein G(I)_G(S)_G(O) subunit gamma-11	2.59	4.28
Gsdma	Gasdermin-A	5.42	5.96
Hsd17b13	17-beta-hydroxysteroid dehydrogenase 13	13.84	2.62
Il17a	Interleukin-17A	10.56	2.54
Il9	Interleukin-9	4.13	2.03
Kcnk10	Potassium channel, subfamily K, member 10	51.93	5.68
Krt24	Keratin, type I cytoskeletal 24	5.06	5.94
Kynu	Kynureninase	7.07	2.28
Mov10l1	RNA helicase Mov10l1	3.29	3.95
Murc	Caveolae-associated protein 4	20.63	3.49
Olr1	Oxidized low-density lipoprotein receptor 1	3.99	2.57
Pdgfc	Platelet-derived growth factor C	3.39	4.28
Pdgfrl	Platelet-derived growth factor receptor-like protein	8.92	4.17

gene_symbol	Full name	Th17/naive	Th17/Treg
Plscr2	Phospholipid scramblase 2	11.53	3.51
Pygo1	Pygopus homolog 1	10.18	5.58
Rbp4	Retinol-binding protein 4	4.16	5.30
Rgs7bp	Regulator of G-protein signaling 7-binding protein	8.59	2.39
Scgn	Secretagogin	4.60	2.85
Slc34a3	Sodium-dependent phosphate transport protein 2C	7.39	4.63
Smarca1	Probable global transcription activator SNF2L1	4.14	4.57
Sntg2	Gamma-2-syntrophin	61.47	2.85
Sphkap	A-kinase anchor protein SPHKAP	7.51	9.45
Spp1	Osteopontin	5.63	2.28
Stac2	SH3 and cysteine-rich domain-containing protein 2	2.10	2.15
Tbx3	T-box transcription factor TBX3	2.11	5.81
Tff1	Trefoil factor 1	4.95	2.55
Thsd7a	Thrombospondin type-1 domain-containing protein 7A	5.50	2.06
Tmc5	Transmembrane channel-like protein 5	2.71	2.08
Tmeff1	Tomoregulin-1	2.96	3.59
Tmeff2	Tomoregulin-2	6.79	2.60
Tshb	Thyrotropin subunit beta	16.17	2.03

gene_symbol	Full name	Th17/naive	Th17/Treg
Ttll8	Protein monoglycylase TTLL8	2.38	3.39
Uroc1	Urocanate hydratase	5.21	4.10
Vax1	Ventral anterior homeobox 1	20.27	2.02
Vsx1	Visual system homeobox 1	2.20	9.40
Wfdc18	WAP four-disulfide core domain protein 18	9.34	7.28
Wnt10b	Protein Wnt-10b	2.48	2.12
Wwtr1	WW domain-containing transcription regulator protein 1	2.36	2.26

Supplementary Figure S1

Separate presentation of female and male mice for the RORgt+ groups presented in original figure 1:

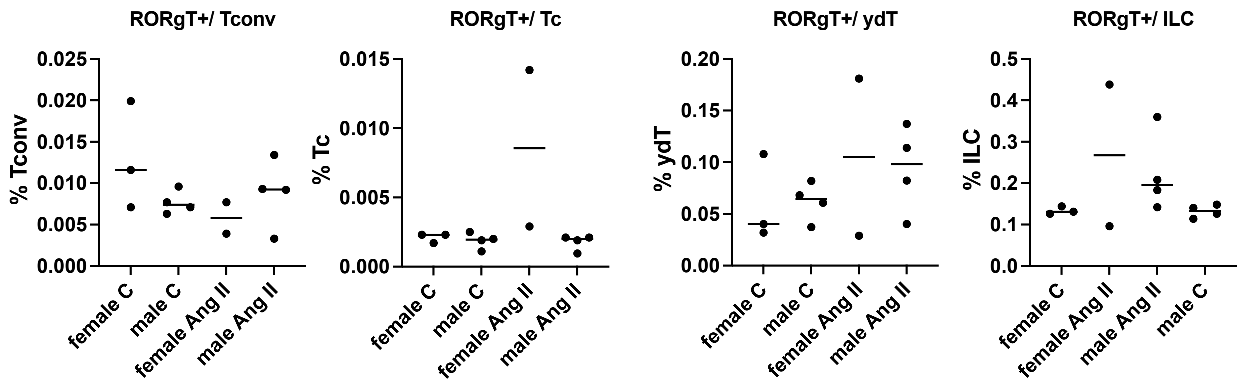

Supplementary Figure S2

Western blot demonstrating the inhibition of Taz by verteporfin.

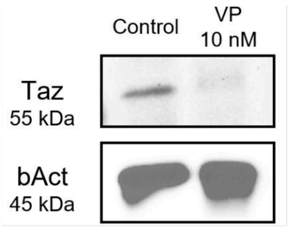

Supplement: Supplementary file 1 [file ijms-26-02143-s001.zip › ijms-3458049-supplementary.pdf]
